# Comparative analysis of pain experience and quality of life in patients with oral lichen planus and burning mouth syndrome

**DOI:** 10.22514/jofph.2026.060

**Published:** 2026-09-12

**Authors:** Ji-Won Kim, Hyo-Jung Jung, Je-Hyun Eom, Jong-Hoon Choi, Jeong-Seung Kwon, Mu-Yeol Cho, Yunwoo Kim, Young-Youn Kim, Hanseung Baek, Hye-Sung Kim, Hyung-Joon Ahn

**Affiliations:** ^1^Department of Orofacial Pain and Oral Medicine, College of Dentistry, Yonsei University, 03722 Seoul, Republic of Korea; ^2^Oral Science Research Institute, College of Dentistry, Yonsei University, 03722 Seoul, Republic of Korea; ^3^Apple Tree Dental Hospital, Apple Tree Medical Foundation, 10387 Goyang-si, Republic of Korea

**Keywords:** Oral lichen planus, Burning mouth syndrome, Pain catastrophizing, Quality of life, Pain interference, Brief Pain Inventory, Chronic oral pain, Cognitive behavioral therapy

## Abstract

**Background**: Oral lichen planus (OLP) and burning mouth syndrome (BMS) are chronic oral conditions associated with persistent pain, but direct comparisons of their pain profiles and contributing factors remain limited. This study compared factors influencing pain perception between OLP and BMS. **Methods**: This cross-sectional study included 46 patients with OLP and 50 with BMS. Pain characteristics, pain catastrophizing, and quality of life were assessed using the Brief Pain Inventory-Korean version, Pain Catastrophizing Scale-Korean version, and a Korean-language Oral Potentially Malignant Disorders Quality of Life questionnaire. Normality was assessed using the Shapiro-Wilk test; Mann-Whitney U or independent *t*-tests were applied accordingly, and effect sizes were calculated using Cohen’s *d*. Pain intensity predictors were identified using stepwise multiple linear regression. **Results**: Patients with BMS reported higher pain intensity (*p* ≤ 0.003, *d* = −1.22 to −0.63) and catastrophizing (*p* = 0.005, *d* = −0.56) than those with OLP, whereas patients with OLP showed greater mastication-related pain interference (*p* = 0.002, *d* = 0.74) and poorer quality of life (*p* = 0.020, *d* = 0.50). Regression identified mastication and rumination in OLP (adjusted *R*^2^ = 0.419) and sleep disturbance, rumination, and magnification in BMS (adjusted *R*^2^ = 0.454) as primary predictors. Age stratification revealed an opposite age–pain relationship: older patients with BMS (>60 years) had higher pain intensity (*p* < 0.001), whereas younger patients with OLP (≤60 years) reported higher pain intensity (*p* = 0.037). Pain interference was more interconnected across life domains in OLP (27 correlations with |*r*| > 0.5) than in BMS (6 correlations). **Conclusions**: OLP and BMS exhibit distinct pain profiles, with OLP characterized by widespread functional interference influenced by mastication-related and cognitive factors, and BMS marked by higher pain intensity with prominent psychological contributors. Condition-specific strategies incorporating cognitive behavioral therapy targeting rumination may improve pain-related outcomes.

## 1. Introduction

Oral lichen planus (OLP) is a chronic, immune-mediated, and inflammatory oral mucosal disease characterized by alternating periods of exacerbation and remission. OLP is considered one of the most prevalent oral mucosal diseases, predominantly affecting middle-aged women, with rare pediatric cases being reported. Clinically, OLP manifests as reticular, atrophic, erosive, plaque-like, papular, or bullous lesions that appear both white and red and occur bilaterally [1]. Among these various OLP subtypes, atrophic and erosive lesions often cause significant pain and discomfort.

Burning mouth syndrome (BMS) is a complex neuropathic chronic pain disorder characterized by a burning sensation in clinically healthy oral mucosa. BMS is more prevalent among peri- and postmenopausal women [2, 3] and is diagnosed following the exclusion of local and systemic etiologies. Its symptoms primarily affect the tongue; however, pain may also occur in the lips, gingiva, buccal mucosa, or palate [4].

Both OLP and BMS are associated with chronic pain that varies with disease severity. The International Association for the Study of Pain defines pain as “an unpleasant sensory and emotional experience associated with, or resembling that associated with, actual or potential tissue damage”, highlighting that pain is a personal experience influenced by biological, psychological, and social factors, irrespective of nociceptor stimulation [5]. Chronic pain is often associated with the impairment or hyperactivation of cognitive and emotional pain-regulating mechanisms, making its management particularly challenging. Psychological factors, such as anxiety and depression, may exacerbate both pain intensity and persistence [6, 7]. Furthermore, anticipation of pain may induce anxiety, fear, and avoidance behaviors, resulting in catastrophizing, a state in which patients perceive their pain as more severe and unmanageable [8]. Pain catastrophizing is defined as “an exaggerated negative mental state regarding actual or anticipated painful experiences” [9] and is further conceptualized as a sense of helplessness, pessimism, and perceived inability to manage pain effectively [10]. Key components of pain catastrophizing include worry, fear, and ruminative focus on pain [11], with stress associated with dental procedures further amplifying catastrophizing-related fear [12]. This maladaptive cognitive pattern is linked to psychological distress, functional impairment [13], and a significant decline in overall quality of life (QoL).

Pain can be categorized, according to its underlying mechanisms, into nociceptive, neuropathic, and nociplastic types [14]. OLP-related pain predominantly involves nociceptive and inflammatory components arising from mucosal lesions, whereas BMS-related pain is primarily characterized by neuropathic and nociplastic mechanisms in the absence of visible pathology. Understanding these mechanistic differences is crucial for implementing appropriate management.

Chronic pain in OLP and BMS has been reported by numerous studies to be strongly associated with psychological comorbidities (*e.g.*, anxiety, depression, and stress) that impair QoL and contribute to disease progression [15, 16, 17]. Although OLP and BMS share chronic oral pain as a common feature, these two conditions differ fundamentally with respect to their pathophysiological mechanisms, suggesting that the nature of pain experience and its effects on daily functioning, as well as psychological factors contributing to pain, may vary substantially between OLP and BMS. Nonetheless, direct comparisons of multifaceted pain characteristics, psychological catastrophizing, and QoL between patients with OLP and BMS remain limited.

Accordingly, the present study aimed to investigate pain intensity, pain interference, pain catastrophizing, and oral health-related QoL in patients with OLP and BMS and to compare factors influencing pain perception between OLP and BMS. We hypothesized that, owing to their distinct pathophysiological mechanisms, OLP and BMS would show different patterns of pain intensity, functional interference, pain catastrophizing, and oral health-related QoL. Through this comparative analysis, this study sought to improve the understanding of condition-specific pain characteristics and contribute to the development of more tailored, and patient-centered treatment approaches.

## 2. Materials and methods

### 2.1 Study design and population

This cross-sectional study enrolled 104 participants (OLP group, n = 51; BMS group, n = 53) admitted to Yonsei University Dental Hospital from March 2024 to September 2024. This study was conducted in accordance with the principles embodied in the Declaration of Helsinki and was approved by the Institutional Review Board of Yonsei University Dental Hospital (IRB no.: 2-2023-0061). Prior to enrollment, written informed consent was obtained from all participants after they had received full explanations about the purpose and protocol of the study. Following the exclusion of patients who had a revised diagnosis (n = 1), received antipsychotic medication (n = 1), withdrew consent (n = 2), and had invalid questionnaire responses (n = 4), data obtained from 96 participants (46 patients with OLP and 50 patients with BMS) were included in the final analysis (Fig. 1).

**Fig. 1.** Flow diagram of the selection of study participants. A total of 104 participants (51 with oral lichen planus (OLP) and 53 with burning mouth syndrome (BMS)) were initially enrolled. Following the exclusion of participants who had a revised diagnosis (n = 1), received antipsychotic medication (n = 1), withdrew consent (n = 2), and had invalid questionnaire responses (n = 4; all from the OLP group), 96 participants (46 with OLP and 50 with BMS) were included in the final analysis.

### 2.2 Inclusion and exclusion criteria

Participants with OLP were diagnosed according to the World Health Organization’s modified diagnostic criteria [18]. The exclusion criteria for OLP were as follows: (i) the presence of other oral mucosal diseases and (ii) cases not histopathologically confirmed as OLP.

Participants with BMS were diagnosed according to the criteria set forth in the International Classification of Headache Disorders, Third Edition. The exclusion criteria for BMS were as follows: (i) iron, folate, zinc, or vitamin B12 deficiency; (ii) a psychiatric diagnosis prior to BMS onset; and (iii) treatment with corticosteroids, antibiotics, or other medications that could cause oral candidiasis.

General exclusion criteria for both the OLP and BMS groups were as follows: (i) systemic conditions, including cancer, head and neck radiotherapy, liver disease, autoimmune disease, and pregnancy; (ii) other conditions that could lead to head and neck pain (*e.g.*, benign or malignant tumors, acute ulcers, dental conditions); and (iii) severe psychological disorders that could interfere with study participation.

### 2.3 General characteristics

Data on the participants’ demographic characteristics, including age, sex, and disease-specific features, were obtained through a self-reported questionnaire. Pain experience, spontaneous pain, stimulus-evoked pain, and oral dryness were dichotomized into “yes”/“no” responses. Pain intensity was determined using a numerical rating scale ranging from 0 (no pain) to 10 (worst pain imaginable) [19].

### 2.4 Brief Pain Inventory–Korean version (BPI-K)

Pain severity, interference, and reduction were evaluated using the BPI-K [20]. Pain severity was assessed by measuring worst, least, average, and current pain over the preceding 24 hours on a scale ranging from 0 to 10. Pain interference was rated across seven domains (namely, activity, mood, mastication, work, relationships, sleep, and enjoyment) on a scale ranging from 0 to 10, with higher scores indicating greater interference. The original “walking” domain was replaced with “mastication” to improve relevance for oral pain conditions [21]. As for pain reduction, the participants were instructed to rate the percentage of pain relief attributable to medication or treatment over the preceding 24 hours on a 0–100% scale.

### 2.5 Pain Catastrophizing Scale–Korean version (PCS-K)

Psychological catastrophizing related to pain was assessed using the PCS-K [11, 22], a 13-item instrument comprising three subscales: helplessness (six items; *e.g.*, “There is nothing I can do to reduce the pain”), rumination (four items; *e.g.*, “I cannot stop thinking about the pain”), and magnification (three items; *e.g.*, “I worry that something serious may happen”). Responses were recorded on a 5-point Likert scale ranging from 0 (not at all) to 4 (always). The total score ranged from 0 to 52, with higher scores indicating greater catastrophizing tendencies.

### 2.6 Oral potentially malignant disorders quality of life (OPMDQoL) questionnaire

QoL related to chronic oral conditions was assessed using the OPMDQoL questionnaire [23], a 20-item instrument that evaluates four domains: difficulty in diagnosis, physical impairment and functional limitations, psychological and social well-being, and treatment effects. Each item was rated on a 5-point Likert scale ranging from 0 (strongly disagree) to 4 (strongly agree), with higher scores indicating poorer QoL. Item 19 (“How satisfied are you with treatment?”) was reverse-scored. The total score ranged from 0 to 80.

### 2.7 Statistical analysis

All statistical analyses were performed using Python version 3.13 (Python Software Foundation, Wilmington, DE, USA) with the SciPy and statsmodels libraries, and statistical significance set at *p* < 0.05.

Normality was examined using the Shapiro-Wilk test for all continuous variables (**Supplementary Table 1**). Because most variables violated normality assumptions (*p* < 0.05), nonparametric methods were primarily employed for group comparisons. Between-group comparisons were performed using the Mann-Whitney U test; when both groups satisfied normality assumptions (*i.e.*, helplessness subscale, “psychological and social well-being” domain, and the total QoL score), an independent *t*-test was applied. Effect sizes were calculated using Cohen’s *d* with pooled standard deviations and were interpreted as follows: trivial, <0.2; small, 0.2–0.5; medium, 0.5–0.8; and large, >0.8. A *post-hoc* power analysis was conducted to verify the adequacy of the sample size for all primary comparisons (**Supplementary Table 2**).

Exploratory analyses included age stratification (≤60 *vs.* >60 years; **Supplementary Table 3**) and examination of the relationship between pain duration and pain intensity (**Supplementary Table 4**).

Multiple linear regression analysis was conducted to identify factors influencing average pain intensity in each group. A stepwise selection method with entry and removal criteria of *p* < 0.05 and *p* > 0.10, respectively, was employed. Model assumptions were verified by examining variance inflation factors (VIFs) for multicollinearity (acceptable if <10), performing the Shapiro-Wilk test for residual normality, visually inspecting residual plots for homoscedasticity, and conducting Cook’s distance analysis for the identification of influential cases (**Supplementary Figs. 1,2**). The independence of residuals was assessed using the Durbin-Watson statistic (acceptable range: 1.5–2.5).

Relationships among pain-related variables were explored using Spearman’s correlation analysis, with correlation matrices presented separately for each group (**Supplementary Tables 5,6** and **Supplementary Figs. 3,4**).

## 3. Results

### 3.1 General characteristics of the study participants

The general characteristics of the study participants are summarized in Table 1. Participants in their 60s composed the highest proportion in both OLP and BMS groups, with the mean age being 58.1 years in the OLP group and 58.8 years in the BMS group. Female patients accounted for 89.1% (41/46) and 94.0% (47/50) in the OLP and BMS groups, respectively. Pain duration was significantly longer in the OLP group than in the BMS group. Stimulus-evoked pain was more prevalent and more intense in the OLP group, whereas spontaneous pain was more frequent and more intense in the BMS group. No significant difference in the proportion of patients with oral dryness was observed between the OLP and BMS groups (65.2% (30/46) *vs.* 70.0% (35/50)).

**Table 1.** General characteristics of the study participants.

Characteristics		OLP (n = 46)	BMS (n = 50)	*p*-value
Age (yr)				
	30–39	2 (4.3)	8 (16.0)	0.111*
	40–49	6 (13.0)	5 (10.0)	
	50–59	15 (32.6)	6 (12.0)	
	60–69	18 (39.1)	22 (44.0)	
	70–79	4 (8.7)	7 (14.0)	
	80–89	1 (2.2)	2 (4.0)	
	Average	58.1 ± 10.6	58.8 ± 13.4	0.373**
Sex				
	Female	41 (89.1)	47 (94.0)	0.388*
	Male	5 (10.9)	3 (6.0)	
Pain duration (yr)		4.9 ± 5.6	1.6 ± 2.5	<0.001**
Stimulus pain				
	No	3 (6.5)	27 (54.0)	<0.001*
	Yes	43 (93.5)	23 (46.0)	
	NRS	4.3 ± 2.7	2.9 ± 3.4	0.030**
Spontaneous pain				
	No	33 (71.7)	0 (0)	<0.001*
	Yes	13 (28.3)	50 (100)	
	NRS	0.7 ± 1.3	4.4 ± 1.6	<0.001**
Dry mouth				
	Yes	30 (65.2)	35 (70.0)	0.617*
	No	16 (34.8)	15 (30.0)	

Data are presented as n (%) for categorical variables and mean ± standard deviation for continuous variables. *Chi-square test; **independent t-test. NRS: numerical rating scale (0–10); OLP: oral lichen planus; BMS: burning mouth syndrome.

### 3.2 Comparison of pain experience and interference between the OLP and BMS groups

Pain experience and interference, as assessed using the BPI-K, significantly differed between the OLP and BMS groups (Table 2). The BMS group exhibited significantly greater pain severity across all subdomains, including worst, least, average, and current pain (all *p* ≤ 0.003), with medium to large effect sizes (Cohen’s *d* = −1.22 to −0.63). With respect to pain interference, scores for the mastication domain were significantly higher in the OLP group (*p* = 0.002, Cohen’s *d* = 0.74); however, scores for the activity, mood, work, relationships, sleep, and enjoyment domains were comparable between the groups. *Post-hoc* power analysis confirmed adequate statistical power (>0.80) for six out of eight primary comparisons (**Supplementary Table 2**). Comparisons for total QoL (power = 0.650) and total pain catastrophizing (power = 0.774) were underpowered; thus, their results should be interpreted with caution.

**Table 2.** Comparison of pain severity and interference between the OLP and BMS groups.

Variable	OLP (n = 46)	BMS (n = 50)	Test	*p*-value	Cohen’s *d*
Pain_Worst	4.3 ± 3.1 (3.0, 2.0–7.0)	6.0 ± 2.2 (6.0, 4.0–8.0)	MWU	0.003*	−0.63 (medium)
Pain_Least	1.0 ± 1.1 (1.0, 0.0–2.0)	2.3 ± 1.5 (2.0, 1.0–3.0)	MWU	<0.001**	−1.00 (large)
Pain_Average	2.8 ± 2.1 (2.5, 1.2–4.0)	4.3 ± 1.7 (4.0, 3.0–5.0)	MWU	<0.001**	−0.80 (medium)
Pain_Current	1.4 ± 1.6 (1.0, 0.0–2.0)	3.7 ± 2.1 (3.0, 2.0–5.0)	MWU	<0.001**	−1.22 (large)
Interference_Activity	2.7 ± 3.3 (1.0, 0.0–5.0)	2.5 ± 2.6 (2.0, 0.0–4.0)	MWU	0.668	0.07 (trivial)
Interference_Mood	5.1 ± 3.5 (5.0, 2.0–8.0)	5.6 ± 2.2 (5.5, 4.0–7.0)	MWU	0.521	−0.20 (small)
Interference_Mastication	3.8 ± 3.3 (3.5, 0.0–7.0)	1.7 ± 2.5 (0.5, 0.0–3.0)	MWU	0.002*	0.74 (medium)
Interference_Work	2.6 ± 3.1 (1.0, 0.0–5.0)	2.1 ± 2.1 (2.0, 0.0–3.0)	MWU	0.964	0.21 (small)
Interference_Relation	3.2 ± 3.3 (2.5, 0.0–5.8)	2.6 ± 2.3 (2.0, 1.0–4.0)	MWU	0.776	0.21 (small)
Interference_Sleep	1.9 ± 3.1 (0.0, 0.0–3.0)	2.6 ± 3.1 (1.0, 0.0–4.5)	MWU	0.054	−0.22 (small)
Interference_Enjoyment	4.3 ± 3.5 (4.0, 1.0–8.0)	5.0 ± 2.5 (5.0, 3.0–7.0)	MWU	0.289	−0.21 (small)

Data are presented as mean ± standard deviation (median, Q1–Q3). *p < 0.01, **p < 0.001. MWU: Mann-Whitney U test; OLP: oral lichen planus; BMS: burning mouth syndrome.

### 3.3 Comparison of pain catastrophizing between the OLP and BMS groups

Pain catastrophizing, as evaluated using the PCS-K, significantly differed between the two groups (Table 3). The BMS group had significantly higher helplessness subscale scores (14.8 ± 6.0 *vs.* 11.5 ± 6.3, *p* = 0.011) and rumination subscale scores (10.3 ± 4.5 *vs.* 7.1 ± 5.0, *p* = 0.001); however, magnification subscale scores showed no significant difference (7.9 ± 3.6 *vs.* 7.1 ± 3.5, *p* = 0.239). The total PCS-K score was significantly higher in the BMS group than in the OLP group (33.0 ± 12.4 *vs.* 25.7 ± 13.6, *p* = 0.005, Cohen’s *d* = −0.56).

**Table 3.** Comparison of pain catastrophizing between the OLP and BMS groups.

Variable	OLP (n = 46)	BMS (n = 50)	Test	*p*-value	Cohen’s *d*
PCS-K_Helplessness	11.5 ± 6.3	14.8 ± 6.0	*t*-test	0.011**	−0.53 (medium)
PCS-K_Magnification	7.1 ± 3.5 (8.0)	7.9 ± 3.6 (8.0)	MWU	0.239	−0.24 (small)
PCS-K_Rumination	7.1 ± 5.0 (6.0)	10.3 ± 4.5 (11.5)	MWU	0.001*	−0.68 (medium)
PCS-K_Total	25.7 ± 13.6 (27.5)	33.0 ± 12.4 (36.0)	MWU	0.005*	−0.56 (medium)

Data are presented as mean ± standard deviation (median), where applicable. Statistical comparisons were performed using the independent t-test or Mann-Whitney U test, as appropriate. *p < 0.01, **p < 0.05. MWU: Mann-Whitney U test; PCS-K: Pain Catastrophizing Scale–Korean version; OLP: oral lichen planus; BMS: burning mouth syndrome.

### 3.4 Comparison of QoL between the OLP and BMS groups

QoL, as assessed using the OPMDQoL questionnaire, significantly differed between the two groups (Table 4). The OLP group scored significantly higher in the “physical impairment and functional limitations” domain than the BMS group (15.5 ± 6.6 *vs.* 10.6 ± 6.4, *p* < 0.001, Cohen’s *d* = 0.75). The total QoL score was significantly higher in the OLP group (42.7 ± 16.5 *vs.* 35.0 ± 14.4, *p* = 0.020, Cohen’s *d* = 0.50), indicating poorer QoL among patients with OLP. Although this 7.7-point difference may represent a clinically meaningful distinction, the statistical power for this comparison was limited (power = 0.650).

**Table 4.** Comparison of quality of life between the OLP and BMS groups.

Variable	OLP (n = 46)	BMS (n = 50)	Test	*p*-value	Cohen’s *d*
Difficulty in diagnosis	7.1 ± 3.7	5.9 ± 3.6	MWU	0.094	0.32 (small)
Physical impairment and functional limitations	15.5 ± 6.6	10.6 ± 6.4	MWU	<0.001*	0.75 (medium)
Psychological and social well-being	16.1 ± 7.6	13.6 ± 5.7	*t*-test	0.072	0.37 (small)
Effect of treatment on daily life	4.0 ± 2.2	4.3 ± 2.0	MWU	0.271	−0.15 (trivial)
Total	42.7 ± 16.5	35.0 ± 14.4	*t*-test	0.020**	0.50 (small)

Data are presented as mean ± standard deviation. Statistical comparisons were performed using the independent t-test or Mann-Whitney U test based on Shapiro-Wilk normality assessment. Item 19 was reverse-scored. Higher scores indicate poorer quality of life. *p < 0.001, **p < 0.05. MWU: Mann-Whitney U test; OLP: oral lichen planus; BMS: burning mouth syndrome.

### 3.5 Age stratification and correlation analyses

Age stratification analysis revealed significant but opposite age effects between the two groups (**Supplementary Table 3**). In the BMS group, patients aged >60 years reported significantly higher average pain intensity than those aged ≤60 years (4.97 ± 1.67 *vs.* 3.35 ± 1.23, *p* < 0.001). Conversely, in the OLP group, younger patients aged ≤60 years reported significantly higher average pain intensity than older patients aged >60 years (3.35 ± 2.17 *vs.* 2.15 ± 1.73, *p* = 0.037), indicating a divergent age–pain relationship between the two conditions. Pain duration did not significantly correlate with pain intensity in both the BMS group (*r* = 0.111, *p* = 0.443) and OLP group (*r* = 0.225, *p* = 0.133) (**Supplementary Table 4**), suggesting that pain duration alone did not determine the severity of pain experience.

Correlation analysis showed distinct patterns between OLP and BMS (**Supplementary Tables 5,6** and **Supplementary Figs. 3,4**). The OLP group exhibited extensive inter-correlations with pain interference domains (27 correlations with |*r*| > 0.5), with particularly strong associations between activity and work (*r* = 0.77), work and sleep (*r* = 0.76), and mastication and work (*r* = 0.75). By contrast, the BMS group showed fewer strong correlations (six correlations with |*r*| > 0.5), with the strongest associations observed between average and current pain (*r* = 0.78) and worst and average pain (*r* = 0.73), suggesting that the effects of pain in BMS were more focal than the pervasive effects observed in OLP.

### 3.6 Factors influencing pain experience in the OLP group

The results of multiple linear regression analysis for factors influencing pain experience in the OLP group are presented in Table 5. Regression diagnostic analysis confirmed the validity of the multivariate model: VIFs were <2.0 for all predictors, residual normality was acceptable (*W* = 0.92, *p* = 0.003), and only one minimally influential case (maximum Cook’s *D* = 0.08) within the acceptable threshold of 4/n was identified in Cook’s distance analysis (**Supplementary Fig. 1**). The model selected using a stepwise method (Durbin-Watson statistic: 1.727) was statistically significant (*F* = 17.194, *p* < 0.001, adjusted *R*^2^ = 0.419). The key predictors were mastication (β = 0.524, *p* < 0.001) and rumination (β = 0.328, *p* = 0.007), with mastication explaining the largest proportion of variance in pain experience based on standardized coefficients.

**Table 5.** Multiple linear regression analysis of factors influencing average pain intensity in the OLP group.

	B	SE	β	*t*	*p*-value
Mastication	0.295	0.065	0.524	4.532	<0.001
Rumination	0.114	0.040	0.328	2.838	0.007

*Stepwise selection method with entry and removal criteria of p < 0.05 and p > 0.10, respectively, was employed. Diagnostic plots are shown in **Supplementary Fig. 1**. R = 0.667, R^2^ = 0.444, adjusted R^2^ = 0.419, F = 17.194, p < 0.001, Durbin-Watson statistic = 1.727. B: unstandardized regression coefficient; SE: standard error; β: standardized regression coefficient; OLP: oral lichen planus.*

### 3.7 Factors influencing pain experience in the BMS group

The results of stepwise multiple linear regression analysis for the predictors of pain intensity in the BMS group are presented in Table 6. Model diagnostics confirmed the independence of residuals (Durbin-Watson statistic: 1.981), acceptable residual normality (*W* = 0.93, *p* = 0.006), VIFs <2.0, and three minimally influential cases (maximum Cook’s *D* = 0.25), all within acceptable thresholds (**Supplementary Fig. 2**). Three variables emerged as significant contributors to the final model (*F* = 14.565, *p* < 0.001, adjusted *R*^2^ = 0.454): sleep interference (β = 0.498, *p* < 0.001), rumination (β = 0.581, *p* < 0.001), and magnification (β = −0.364, *p* = 0.014). Among these predictors, rumination showed the strongest association with pain intensity, followed by sleep disturbance. Notably, magnification exhibited an unexpected inverse relationship with pain experience (*B* = −0.173), indicating that higher magnification scores were associated with lower reported pain intensity. This counterintuitive finding is further explored in the succeeding Discussion section.

**Table 6.** Multiple linear regression analysis of factors influencing average pain intensity in the BMS group.

	B	SE	β	*t*	*p*-value
Sleep	0.267	0.059	0.498	4.543	<0.001
Rumination	0.218	0.053	0.581	4.100	<0.001
Magnification	−0.173	0.068	−0.364	−2.564	0.014

*Stepwise selection method with entry and removal criteria of p < 0.05 and p > 0.10, respectively, was employed. Diagnostic plots are shown in **Supplementary Fig. 2**. R = 0.698, R^2^ = 0.487, adjusted R^2^ = 0.454, F = 14.565, p < 0.001, Durbin-Watson statistic = 1.981. B: unstandardized regression coefficient; SE: standard error; β: standardized regression coefficient; BMS: burning mouth syndrome.*

## 4. Discussion

This study compared pain experience, interference, catastrophizing, and QoL between patients with OLP and BMS to identify factors influencing pain perception in these two chronic oral pain conditions. Pain management is central to OLP and BMS treatment and requires a multifaceted assessment encompassing not only the presence and intensity of pain, but also its effects on daily functioning and psychological well-being.

Both groups showed a female predominance with a mean age of approximately 58 years, consistent with the findings of previous epidemiological studies reporting female proportions of 65.9% [24] and 76.7% [25] for OLP and 84.0% [26] and 83.9% [27] for BMS, with a mean age of approximately 50 years. Pain duration was significantly longer in the OLP group; this was an expected finding, given that OLP is an autoimmune-related condition requiring longer-term management [28]. In the OLP group, stimulus-evoked pain was more prevalent and more intense. In the BMS group, all 50 patients experienced spontaneous pain, reflecting the defining characteristic of this condition [29].

The BPI-K results indicated significantly higher overall pain severity scores in patients with BMS than in those with OLP, consistent with the predominance of spontaneous pain in BMS. Patients with OLP exhibited significantly greater pain interference with mastication (*p* = 0.002); however, activity, mood, work, relationships, sleep, and enjoyment domains were comparable between the groups. These findings align with the results of a previous study showing that oral mucosal lesions in OLP caused discomfort during food mastication and that patients with BMS may experience temporary pain reduction through mastication [30]. The divergent patterns of pain interference between OLP and BMS highlight the importance of considering both functional and psychological factors when developing condition-specific pain management strategies. Of note, the BMS diagnostic criteria applied in this study required the presence of chronic pain symptoms (lasting for >2 hours/day over a period of several months), and patients with OLP were enrolled based on clinical and histopathological confirmation, irrespective of symptomatic status. Consequently, a proportion of patients with OLP might have been asymptomatic or minimally symptomatic at enrollment, possibly contributing to the lower mean pain severity scores observed in the OLP group. Out of 46 patients with OLP who were included in the final analysis, only 13 (28.3%) experienced spontaneous oral pain at enrollment; in contrast, all 50 patients with BMS presented with spontaneous pain as a defining diagnostic criterion. This structural asymmetry in the enrollment criteria represents an inherent limitation in the direct comparison of pain intensity between the two groups and should be considered when interpreting between-group differences in pain severity scores.

The divergent patterns of oral functional impairment observed in the present study have important implications for clinical assessment and management in oral medicine practice. The significantly greater mastication interference among patients with OLP (*p* = 0.002) reflects the direct impact of mucosal lesions on oral function, whereas the predominance of spontaneous pain among all 50 patients with BMS underscores the neuropathic nature of this condition. Patients with BMS often report paradoxical improvement during eating; notably, a recent retrospective study reported pain relief in 69.7% of patients with BMS and identified eating, chewing, or sucking as the primary alleviating factor in 91.6% [30]. This phenomenon may be attributed to the activation of large-diameter mechanoreceptive fibers during mastication, which modulate nociceptive transmission through the gate control mechanism originally described by Melzack and Wall [31], and is further supported by experimental evidence suggesting the suppression of nociceptive responses by hard-food mastication [32]. These contrasting oral functional profiles underscore the necessity of incorporating standardized evaluations of both stimulus-evoked and spontaneous pain components into routine clinical assessment because relying on a single pain dimension may underestimate the functional burden in OLP or the psychological burden in BMS.

The PCS-K results indicated significantly greater pain catastrophizing in patients with BMS than in those with OLP (*p* = 0.005, Cohen’s *d* = −0.56), suggesting the stronger influence of psychological factors on pain experience in BMS. This finding aligns with the results of a previous study, which reported that patients with fibromyalgia—a chronic generalized pain condition characterized by widespread muscle pain, fatigue, and sleep disturbance lasting for >3 months without a specific etiology—experienced greater psychological catastrophizing when exposed to painful stimuli [33]. Given the hypothesized similarities between fibromyalgia and BMS with respect to neuropathic pain mechanisms [34], increased catastrophizing among patients with BMS in the present study is consistent with this framework. Nevertheless, the statistical power for this comparison (power = 0.774) was limited; thus, these findings should be confirmed in larger samples. Notably, rumination was identified as a common factor with a substantial impact on pain experience in both conditions, suggesting that cognitive behavioral therapy (CBT) targeting ruminative thinking may serve as an effective strategy for managing chronic pain in both OLP and BMS.

The OPMDQoL questionnaire results indicated that patients with OLP experienced significantly greater physical impairment and functional limitations than those with BMS (*p* < 0.001, Cohen’s *d* = 0.75). Additionally, patients with OLP tended to exhibit poorer psychological and social well-being; however, this difference did not reach statistical significance (*p* = 0.072). The total QoL score was significantly higher in the OLP group (*p* = 0.020, Cohen’s *d* = 0.50), indicating poorer overall QoL despite lower pain intensity.

From an oral health perspective, particular attention should be directed toward the significantly poorer overall QoL observed in patients with OLP, despite their lower pain intensity scores. The 7.7-point difference in total OPMDQoL questionnaire scores between the two groups suggests a clinically meaningful distinction; however, the statistical power for this comparison was limited (power = 0.650), and this result should be confirmed in larger cohorts. In OLP, the impact on QoL extends beyond pain intensity to encompass broader concerns related to oral function, dietary restrictions, and psychosocial burden of living with a potentially malignant oral condition. The “physical impairment and functional limitations” domain, which includes difficulties in eating, speaking, and oral hygiene, was the primary driver of reduced QoL in OLP. These results emphasize the need for comprehensive QoL assessment as a routine component of OLP management, rather than relying solely on clinical severity scores or pain measures.

Multiple linear regression analysis identified mastication and rumination as the primary predictors of pain experience in the OLP group and sleep disturbance, rumination, and magnification as the main contributing factors in the BMS group. The prominent role of mastication in the OLP group is supported by prior research [35], whereas the significant contribution of sleep disturbance to pain experience in the BMS group is consistent with previous findings [36, 37]. Rumination had a significant effect in both groups, with increased ruminative tendencies associated with greater pain experience. This is consistent with previous findings that higher pain catastrophizing is associated with greater pain intensity and that rumination increases pain intensity in chronic pain patients [38, 39]. These findings support the utility of CBT focused on reducing ruminative thinking as a pain management approach for both conditions. An unexpected negative association was observed between magnification and pain experience in the BMS group, which contradicts previous findings linking higher catastrophizing to greater pain intensity. Several explanations may account for this finding. First, the cross-sectional design of this study did not capture the timing and duration of pain symptom onset; patients with longstanding BMS may have developed heightened awareness of potential pain consequences (high magnification) while simultaneously achieving partial psychological adaptation to their chronic symptoms, resulting in lower reported pain intensity. Second, the regression model may have been subject to statistical suppression, as magnification was entered alongside correlated predictors including rumination and sleep disturbance. Third, patients with higher magnification may engage in more proactive pain-avoidance behaviors, such as dietary modifications or stimulus avoidance, thereby reducing their actual pain exposure. It should be emphasized that this finding is exploratory in nature, given the modest sample size of the BMS group, and may reflect a statistical suppression effect rather than a true negative relationship: when magnification shares variance with correlated predictors such as rumination and sleep disturbance, partialling out this shared variance can artificially reverse the sign of the magnification coefficient in the regression model. Accordingly, this result should not be interpreted as evidence that magnification reduces pain, but rather as a statistical artifact warranting cautious interpretation. Future studies employing longitudinal designs, larger samples, and mediation analyses are needed to clarify the nature of this counterintuitive relationship.

Age stratification analysis revealed significant but opposite age effects in the two groups: older patients with BMS (>60 years) reported significantly higher pain intensity than younger patients (*p* < 0.001), whereas younger patients with OLP (≤60 years) reported significantly higher pain intensity than their older counterparts (*p* = 0.037). The age-dependent increase in pain severity observed in BMS is consistent with findings that neuropathic pain conditions may worsen with age-related neurological changes. In contrast, the higher pain in younger patients with OLP may reflect greater disease activity or heightened pain sensitivity in this subgroup; it is conceivable that this pattern is partly driven by a higher prevalence of erosive or atrophic OLP subtypes among younger patients, given that these subtypes are typically associated with more severe pain than the reticular forms, although subtype data were not available in the present study to confirm this hypothesis. Further investigation with longitudinal designs and prospective subtype recording is warranted.

This study has some limitations. First, the cross-sectional design did not account for the timing of participants’ clinical visits or the temporal dynamics of their pain experience, constraining the ability to establish causal relationships among the analyzed variables. Second, the two diagnostic groups were subject to discrepant inclusion criteria regarding pain: patients with BMS were diagnosed based on the presence of chronic oral pain as a defining feature, whereas patients with OLP were enrolled based on diagnostic confirmation alone, without a minimum pain threshold. Such mismatch might have introduced selection bias via the inclusion of patients with OLP with minimal or absent pain, as reflected by the finding that only 13 (28.3%) out of 46 patients with OLP experienced spontaneous pain at enrollment; this should be considered when interpreting between-group differences in pain severity. Third, the sample size was determined based on the available patient population during the study period rather than through an *a priori* power calculation. Furthermore, *post-hoc* power analysis revealed that two primary comparisons namely, total pain catastrophizing (power = 0.774) and total QoL (power = 0.650) were underpowered, implying that the sample size may have been insufficient to reliably detect moderate effect sizes for these outcomes. Future studies with larger adequately powered sample sizes should be conducted to confirm these findings. Fourth, this study was conducted at a single tertiary institution, potentially limiting the generalizability of its findings. Further multicenter studies should be performed to validate the findings across diverse clinical settings. Fifth, the OLP group was not stratified by clinical subtype (*e.g.*, reticular, atrophic, and erosive). Given that erosive and atrophic OLP subtypes are associated with greater pain and psychological burden, the clinical heterogeneity within the OLP cohort in the present study may have contributed to variability in pain outcomes. Future studies should classify OLP according to dominant subtypes to further clarify the differential impact of lesion type on pain experience and QoL. Sixth, the small number of male participants (five in the OLP group and three in the BMS group) precluded meaningful sex-stratified analyses. Considering the known sex differences in chronic pain perception, future studies with a balanced sex distribution are required to investigate potential sex-specific effects.

Despite these limitations, this study is significant because it represents the first direct comparison of multifaceted pain experience, pain catastrophizing, and QoL between patients with OLP and BMS. The findings indicated that pain characteristics and their influencing factors differed distinctly between OLP and BMS. On the one hand, patients with BMS reported higher pain intensity and greater pain catastrophizing driven primarily by sleep disturbance, rumination, and magnification. On the other hand, patients with OLP experienced greater mastication-related discomfort and poorer overall QoL, with pain interference being pervasively interconnected across multiple life domains. Age stratification analysis revealed an opposite age–pain relationship between the two conditions: older patients with BMS exhibited significantly increased pain severity, whereas younger patients with OLP showed higher pain intensity.

## 5. Conclusions

From a clinical management perspective, the findings of the present study support a differentiated therapeutic approach. For OLP, interventions should prioritize the restoration of comfortable oral function through the administration of topical anti-inflammatory agents targeting mucosal lesions, dietary counseling to minimize mastication-related discomfort, and regular monitoring of broader functional impact on daily activities. For BMS, management should address prominent psychological contributors while paying particular attention to sleep hygiene interventions and age-appropriate pain management strategies, especially for patients aged >60 years with significantly increased pain levels. For both OLP and BMS, CBT should be integrated into standard oral medicine practice, given the significant role of rumination as a predictor of pain experience. Specifically, CBT techniques targeting ruminative thinking patterns (*e.g.*, cognitive restructuring, mindfulness-based approaches, and attention-diversion strategies) may be effective adjunctive treatments that complement conventional pharmacological management. Notably, between-group comparisons for total pain catastrophizing (power = 0.774) and total QoL (power = 0.650) were underpowered; therefore, these findings should be regarded as preliminary, pending confirmation in larger adequately powered cohorts. Future studies with larger sample sizes should be conducted to confirm these and other findings of the present study. In particular, longitudinal studies should be performed to clarify causal relationships and formulate more targeted pain management recommendations.

## Supplementary Material

Supplementary material associated with this article can be found, in the online version, at https://files.jofph.com/files/article/2098295288135335936/attachment/Supplementary%20material.docx.


