# Supplementary material for: Comparative analysis of pain experience and quality of life in patients with oral lichen planus and burning mouth syndrome
Source: J Oral Facial Pain Headache. 2026 Sep 12;40(5):54–64. doi: 10.22514/jofph.2026.060 (PMC13678553; doi:10.22514/jofph.2026.060)
Supplement: Supplementary file 1 [file Supplementary-material.docx]

Supplementary material

Supplementary Table 1. Shapiro-Wilk normality test results for all continuous variables.

| Variable | Group | N | Mean | SD | Median | IQR | Shapiro-W | *p*-value | Normal |
| --- | --- | --- | --- | --- | --- | --- | --- | --- | --- |
| Pain_Worst | BMS | 50 | 6 | 2.16 | 6 | 4.0–8.0 | 0.9331 | 0.0073 | No |
| Pain_Worst | OLP | 46 | 4.3 | 3.14 | 3 | 2.0–7.0 | 0.9204 | 0.0039 | No |
| Pain_Least | BMS | 50 | 2.3 | 1.54 | 2 | 1.0–3.0 | 0.8951 | 0.0003 | No |
| Pain_Least | OLP | 46 | 0.96 | 1.07 | 1 | 0.0–2.0 | 0.7927 | <0.0010 | No |
| Pain_Average | BMS | 50 | 4.32 | 1.7 | 4 | 3.0–5.0 | 0.9084 | 0.0009 | No |
| Pain_Average | OLP | 46 | 2.83 | 2.06 | 2.5 | 1.2–4.0 | 0.9171 | 0.003 | No |
| Pain_Current | BMS | 50 | 3.7 | 2.11 | 3 | 2.0–5.0 | 0.9318 | 0.0065 | No |
| Pain_Current | OLP | 46 | 1.41 | 1.59 | 1 | 0.0–2.0 | 0.8256 | <0.0010 | No |
| Interference_Activity | BMS | 50 | 2.46 | 2.62 | 2 | 0.0–4.0 | 0.8563 | <0.0010 | No |
| Interference_Activity | OLP | 46 | 2.65 | 3.26 | 1 | 0.0–5.0 | 0.7832 | <0.0010 | No |
| Interference_Mood | BMS | 50 | 5.64 | 2.17 | 5.5 | 4.0–7.0 | 0.9687 | 0.2057 | Yes |
| Interference_Mood | OLP | 46 | 5.07 | 3.48 | 5 | 2.0–8.0 | 0.9017 | 0.0009 | No |
| Interference_Mastication | BMS | 50 | 1.66 | 2.49 | 0.5 | 0.0–3.0 | 0.7149 | <0.0010 | No |
| Interference_Mastication | OLP | 46 | 3.78 | 3.25 | 3.5 | 0.0–7.0 | 0.8929 | 0.0005 | No |
| Interference_Work | BMS | 50 | 2.08 | 2.06 | 2 | 0.0–3.0 | 0.8637 | <0.0010 | No |
| Interference_Work | OLP | 46 | 2.63 | 3.12 | 1 | 0.0–5.0 | 0.8097 | <0.0010 | No |
| Interference_Relation | BMS | 50 | 2.62 | 2.34 | 2 | 1.0–4.0 | 0.901 | 0.0005 | No |
| Interference_Relation | OLP | 46 | 3.22 | 3.33 | 2.5 | 0.0–5.8 | 0.8409 | <0.0010 | No |
| Interference_Sleep | BMS | 50 | 2.58 | 3.15 | 1 | 0.0–4.5 | 0.7816 | <0.0010 | No |
| Interference_Sleep | OLP | 46 | 1.89 | 3.07 | 0 | 0.0–3.0 | 0.6689 | <0.0010 | No |
| Interference_Enjoyment | BMS | 50 | 4.98 | 2.54 | 5 | 3.0–7.0 | 0.9617 | 0.1042 | Yes |
| Interference_Enjoyment | OLP | 46 | 4.35 | 3.48 | 4 | 1.0–8.0 | 0.8934 | 0.0005 | No |

Normality was assessed separately for OLP and BMS groups. Variables with *p* < 0.05 were considered to violate normality assumptions, justifying the use of non-parametric statistical methods (Mann-Whitney U tests) for between-group comparisons. N: sample size; SD: standard deviation; IQR: interquartile range; Shapiro-W: Shapiro-Wilk test statistic; OLP: oral lichen planus; BMS: burning mouth syndrome.

Supplementary Table 2. *Post-hoc* power analysis for primary between-group comparisons.

| Variable | Cohen_*d* | N_OLP | N_BMS | Power |
| --- | --- | --- | --- | --- |
| Pain_Worst | −0.63 | 46 | 50 | 0.8626 |
| Pain_Least | −1 | 46 | 50 | 0.998 |
| Pain_Average | −0.8 | 46 | 50 | 0.9723 |
| Pain_Current | −1.22 | 46 | 50 | 1 |
| PCS-K Total | −0.56 | 46 | 50 | 0.7743 |
| PCS-K Rumination | −0.68 | 46 | 50 | 0.9089 |
| QoL_Total | 0.5 | 46 | 44 | 0.65 |
| Interference_Mastication | 0.74 | 46 | 50 | 0.9479 |

Statistical power was calculated based on observed effect sizes (Cohen’s *d*), actual sample sizes, and significance level (α = 0.05) for two-tailed independent *t*-tests. Power values >0.80 indicate adequate statistical power to detect clinically meaningful differences.

OLP: oral lichen planus; BMS: burning mouth syndrome; PCS-K: Pain Catastrophizing Scale-Korean version; QoL: quality of life.

Supplementary Table 3. Age stratification analysis comparing pain intensity between younger (≤60 years) and older (>60 years) participants within each disease group.

| Group | Variable | Age ≤60_n | Age ≤60_Mean | Age ≤60_SD | Age >60_n | Age >60_Mean | Age >60_SD | *p*_value |
| --- | --- | --- | --- | --- | --- | --- | --- | --- |
| BMS | Average | 20 | 3.35 | 1.23 | 30 | 4.97 | 1.67 | <0.001 |
| OLP | Average | 26 | 3.35 | 2.17 | 20 | 2.15 | 1.73 | 0.037 |

Data are presented as n for sample size and mean ± standard deviation for pain intensity. Statistical comparisons were performed using Mann-Whitney U tests. Significant age effects were observed in both groups: older BMS patients reported higher pain intensity (*p* < 0.001), while younger OLP patients reported higher pain intensity (*p* = 0.037), suggesting opposite age-pain relationships between the two conditions.

OLP: oral lichen planus; BMS: burning mouth syndrome; SD: standard deviation.

Supplementary Table 4. Spearman correlation analysis between pain duration (in months) and average pain intensity in OLP and BMS groups.

| Group | Variable | n | Spearman_*r* | *p*_value |
| --- | --- | --- | --- | --- |
| BMS | Average | 50 | 0.111 | 0.443 |
| OLP | Average | 46 | 0.225 | 0.133 |

No significant correlations were found in either group, indicating that disease chronicity does not predict pain severity. *r*: Spearman’s correlation coefficient; n: number of participants with complete data for both variables; OLP: oral lichen planus; BMS: burning mouth syndrome.

**Supplementary Fig. 1. Regression diagnostic plots for the OLP multiple regression model (Table 5).** Left panel: Residuals *vs*. fitted values plot to assess homoscedasticity; the random scatter around the horizontal line at zero indicates acceptable homoscedasticity. Right panel: Q-Q (quantile-quantile) plot to assess normality of residuals; points closely following the diagonal line indicate approximately normal distribution. One potential influential case was identified but remained within acceptable thresholds (Cook’s distance <0.1). OLP: oral lichen planus.

**Supplementary Fig. 2. Regression diagnostic plots for the BMS multiple regression model (Table 6).** Left panel: Residuals *vs*. fitted values plot showing acceptable homoscedasticity with no clear pattern in residual distribution. Right panel: Q-Q plot indicating approximately normal distribution of residuals. Three potential influential cases were identified but remained within acceptable thresholds (Cook’s distance <0.3), confirming model validity. BMS: burning mouth syndrome; Q-Q plot: quantile-quantile plot.

Supplementary Table 5. Spearman correlation matrix for the OLP group.

|  | Worst | Least | Average | Current | Activity | Mood | Mastication | Work | Relation | Sleep | Enjoyment |
| --- | --- | --- | --- | --- | --- | --- | --- | --- | --- | --- | --- |
| Worst | 1.00 | 0.37 | 0.55 | 0.48 | 0.44 | 0.59 | 0.55 | 0.49 | 0.44 | 0.19 | 0.40 |
| Least | 0.37 | 1.00 | 0.51 | 0.74 | 0.43 | 0.27 | 0.44 | 0.46 | 0.41 | 0.44 | 0.28 |
| Average | 0.55 | 0.51 | 1.00 | 0.62 | 0.32 | 0.42 | 0.49 | 0.37 | 0.37 | 0.25 | 0.31 |
| Current | 0.48 | 0.74 | 0.62 | 1.00 | 0.50 | 0.38 | 0.60 | 0.56 | 0.56 | 0.39 | 0.45 |
| Activity | 0.44 | 0.43 | 0.32 | 0.50 | 1.00 | 0.57 | 0.62 | 0.77 | 0.66 | 0.65 | 0.65 |
| Mood | 0.59 | 0.27 | 0.42 | 0.38 | 0.57 | 1.00 | 0.61 | 0.47 | 0.57 | 0.38 | 0.63 |
| Walking | 0.55 | 0.44 | 0.49 | 0.60 | 0.62 | 0.61 | 1.00 | 0.75 | 0.61 | 0.60 | 0.55 |
| Work | 0.49 | 0.46 | 0.37 | 0.56 | 0.77 | 0.47 | 0.75 | 1.00 | 0.69 | 0.76 | 0.47 |
| Relation | 0.44 | 0.41 | 0.37 | 0.56 | 0.66 | 0.57 | 0.61 | 0.69 | 1.00 | 0.62 | 0.60 |
| Sleep | 0.19 | 0.44 | 0.25 | 0.39 | 0.65 | 0.38 | 0.60 | 0.76 | 0.62 | 1.00 | 0.54 |
| Enjoyment | 0.40 | 0.28 | 0.31 | 0.45 | 0.65 | 0.63 | 0.55 | 0.47 | 0.60 | 0.54 | 1.00 |

The matrix displays correlations among pain severity measures (worst, least, average, current) and pain interference domains (activity, mood, walking/mastication, work, relationships, sleep, enjoyment). Correlations with |*r*| > 0.5 are considered strong and indicate substantial inter-relationships among pain interference domains. Visual representation is provided in **Supplementary Fig. 3**.

Supplementary Table 6. Spearman correlation matrix for the BMS group. The matrix displays correlations among pain severity measures (worst, least, average, current) and pain interference domains (activity, mood, walking/mastication, work, relationships, sleep, enjoyment).

|  | Worst | Least | Average | Current | Activity | Mood | Mastication | Work | Relation | Sleep | Enjoyment |
| --- | --- | --- | --- | --- | --- | --- | --- | --- | --- | --- | --- |
| Worst | 1.00 | 0.36 | 0.73 | 0.49 | 0.24 | 0.41 | 0.15 | 0.12 | 0.34 | 0.27 | 0.48 |
| Least | 0.36 | 1.00 | 0.46 | 0.51 | 0.14 | 0.13 | 0.09 | 0.12 | 0.23 | 0.03 | 0.29 |
| Average | 0.73 | 0.46 | 1.00 | 0.78 | 0.27 | 0.40 | 0.18 | 0.08 | 0.33 | 0.40 | 0.38 |
| Current | 0.49 | 0.51 | 0.78 | 1.00 | 0.22 | 0.22 | 0.06 | 0.06 | 0.37 | 0.33 | 0.40 |
| Activity | 0.24 | 0.14 | 0.27 | 0.22 | 1.00 | 0.17 | 0.41 | 0.53 | 0.38 | 0.32 | 0.24 |
| Mood | 0.41 | 0.13 | 0.40 | 0.22 | 0.17 | 1.00 | 0.22 | 0.22 | 0.39 | 0.21 | 0.63 |
| Walking | 0.15 | 0.09 | 0.18 | 0.06 | 0.41 | 0.22 | 1.00 | 0.66 | 0.34 | 0.32 | 0.09 |
| Work | 0.12 | 0.12 | 0.08 | 0.06 | 0.53 | 0.22 | 0.66 | 1.00 | 0.41 | 0.20 | 0.08 |
| Relation | 0.34 | 0.23 | 0.33 | 0.37 | 0.38 | 0.39 | 0.34 | 0.41 | 1.00 | 0.11 | 0.47 |
| Sleep | 0.27 | 0.03 | 0.40 | 0.33 | 0.32 | 0.21 | 0.32 | 0.20 | 0.11 | 1.00 | 0.26 |
| Enjoyment | 0.48 | 0.29 | 0.38 | 0.40 | 0.24 | 0.63 | 0.09 | 0.08 | 0.47 | 0.26 | 1.00 |

Fewer strong correlations (|*r*| > 0.5) were observed compared to the OLP group, suggesting more focal impact of pain. Visual representation is provided in **Supplementary Fig. 4**.

**Supplementary Fig. 3. Correlation heatmap for the OLP group.** Visual representation of the Spearman correlation matrix (**Supplementary Table 5**) displaying relationships among pain variables. Color intensity indicates correlation strength: red for positive correlations, blue for negative correlations, with darker colors representing stronger correlations. The extensive network of strong correlations (|*r*| > 0.5) demonstrates the pervasive impact of pain across multiple life domains in OLP patients. OLP: oral lichen planus.

**Supplementary Fig. 4. Correlation heatmap for the BMS group.** Visual representation of the Spearman correlation matrix (**Supplementary Table 6**) displaying relationships among pain variables. Color scheme is identical to **Supplementary Fig. 3**. The sparser pattern of strong correlations compared to **Supplementary Fig. 3** indicates more focal impact of pain in BMS despite higher pain intensity, possibly reflecting the neuropathic nature of pain without visible pathology. BMS: burning mouth syndrome.
